# Recent Advances in Printed Capacitive Sensors

**DOI:** 10.3390/mi11040367

**Published:** 2020-04-01

**Authors:** Almudena Rivadeneyra, Juan Antonio López-Villanueva

**Affiliations:** Departamento de Electrónica y Tecnología de Computadores, ETSIIT Universidad de Granada, E-18071 Granada, Spain; arivadeneyra@ugr.es

**Keywords:** interdigitated electrodes, inkjet printing, roll-to-roll, screen printing, spray deposition

## Abstract

In this review paper, we summarize the latest advances in the field of capacitive sensors fabricated by printing techniques. We first explain the main technologies used in printed electronics, pointing out their features and uses, and discuss their advantages and drawbacks. Then, we review the main types of capacitive sensors manufactured with different materials and techniques from physical to chemical detection, detailing the main substrates and additives utilized, as well as the measured ranges. The paper concludes with a short notice on status and perspectives in the field.

## 1. Introduction

A sensor can be defined as a device which reacts with a signal when it receives a stimulus from its surroundings. The generated signal in response to the stimulus is usually converted into an electrical one [1,2]. The importance of sensors in our society is crucial, from the correct functioning of complex electronic systems to the extraction of valuable information of our environment, thus contributing to our wellness [3]. 

We can find many different classifications for sensors. The most common ones are summarized [4] in Table 1.

In this paper, we focus our attention on capacitive sensors. A capacitor is a device capable of storing energy when polarized by an electric field generated by voltage applied across its terminals [5]. The simplest capacitive structure consists of a dielectric material among parallel plates (PP). The capacitance indicates the ability of these two PPs to hold such charge.

Capacitive sensors associate the change in the capacitance between two or more conductors in a dielectric environment to the variation in the parameter of interest [6]. In general, when a capacitive sensor is employed, the sensing surface of the probe is often the electrified plate and the target is the other plate. The sensor front-end constantly varies the voltage on the sensing surface. This voltage is known as the excitation voltage. The necessary current to modify this voltage is obtained by the circuit and determines the capacitance between the probe and the target. Alternatively, a fixed current is applied into the capacitor and the originated voltage is measured [7].

Capacitive sensors have been widely utilized for many years in many different applications. Some examples are detailed below [6]:

- Measurement: pressure (changes in pressure can be directly detected as a dielectric constant change or loss-tangent change), flow (capacitive flowmeters can measure the displacement directly or convert pressure into displacement through a diaphragm), chemical concentration (capacitive chemical sensors can detect the analyte content directly as a dielectric constant change).
- Proximity sensing: personnel detection (safety opening when a person is too far or too close), vehicle detection.
- Communication: Radiofrequency propagation in the near field can be measured by a receiver with a capacitive antenna.

One possible classification of capacitive sensors can be made according to the sensitive variable, that is, which part of a specific capacitive structure is responsible for its behaviour as a sensor. In this regard, we can distinguish two categories [8]:

- Electrical properties of the constitutive materials. The most common one is a dependence of the electrical permittivity of the dielectric with the variable of interest.
- Displacement of the electrodes. A physical displacement in any of the electrode axes produced by the sensing variable.

**Figure 1:**
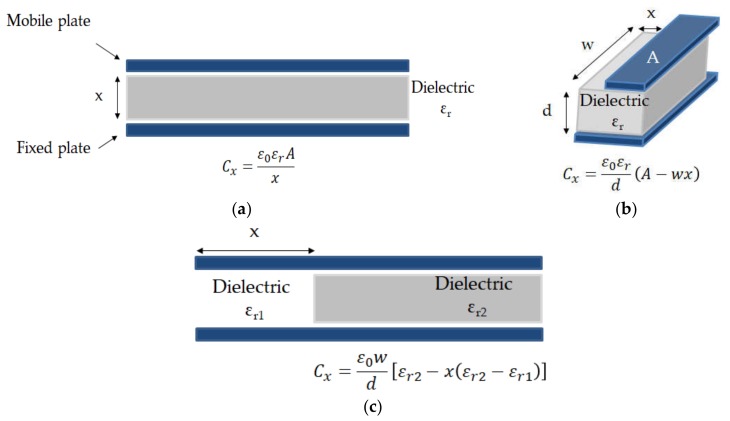
Variables in capacitive sensors: (**a**) Distance (*x*) between plates; (**b**) Capacitor area (electrode moving in *x* direction); (**c**) Dielectric properties.

The simplest capacitive structure is the PP capacitor that consists of two electrodes separated by a dielectric material. Figure 1 illustrates the different capacitive sensors that can be found with a PP capacitor.

Capacitive sensors are normally preferred because of their lower power consumption and faster response with respect to resistive sensors [9,10]. With respect to the integration of capacitive sensors in electronic circuits, there are many different circuits’ configurations to measure capacitance. Among the alternatives, one of the most used is the charge transfer [11] and the current sense amplifier [12]. Another one is based on an alternating current bridge [13]. In general, most of these circuits require a reference capacitor to determine the sensing capacitance.

In this review, we are going to explore the advances in capacitive sensors developed with a bunch of technologies known as printed electronics (PE). These techniques employ common printing equipment suitable for the definition of patterns on materials to create electronics on different substrates [14]. The paper is structured as follows. In Section 2, we describe the main printing techniques utilized for electronics. Then, we detail the latest advances in physical capacitive sensors in Section 3 and we illustrate the progress in printed chemical capacitive sensors in Section 4. Finally, the main conclusions and future perspectives in the field of printed capacitive sensors are outlined in Section 5.

## 2. Fabrication Technologies: Printed Electronics

Among the emerging technologies for the manufacturing of sensors, PE has aroused much attraction because of the extra features it can offer with respect to conventional fabrication processes. Some of these characteristics are the possibility of developing electronics in virtually any surface, including rigid and non-rigid substrates; the production at large-scale, resulting in cost-effective devices where the inclusion of new manufacturing steps or/and materials is almost direct; the likelihood of employing novel materials with outstanding attributes, such as stretchability, biodegradability, and biocompatibility [15,16]. In this section, we summarize the main fabrication technologies in PE, highlighting their main features. To do that, we use a standard classification based on the contact between the deposited material and the substrate while manufacturing.

### 2.1. Non-Contact Technologies

This group of non-contact solutions includes those where only the deposited material is in direct contact with the substrate. The non-contact technologies have the advantage with respect to the contact ones that the substrate is only in contact with the deposited materials. This feature decreases the likelihood of contamination and of substrate deterioration. Furthermore, the alignment of layers is more accurate, which is particularly important in multilayered devices and system integration. Another advantage of this bunch of techniques is the fact that no physical mask is required to define the desired layout but only a digital file. This characteristic not only reduces the switching process without any additional cost but also facilitates the redesign in terms of time and costs [17]. Nevertheless, the non-contact technologies have also some disadvantages like the throughput when fabricating at large-scale [18].

All kind of substrates, from metals and glass to rubbers and polymers, can be utilized, including those which can only be processed with low temperatures and suffer from risk to be deformed or damage when they are exposed to thermal stresses and high temperatures [16]. In this section, we outline the main non-contact printing technologies.

#### 2.1.1. Inkjet Printing

Inkjet printing has attracted a lot of attention in the development of new flexible electronic applications [19]. In inkjet printing, the desired layout is stored in digital format which is directly transferred to the substrate by the material droplets generated in the printer nozzles without the use of masks and the need of contact between printer and substrate.

The droplets can be fluid or powder, proteins or minerals [20,21], conductive polymers [22,23], or nanoparticles [24,25] among a wide range of materials, which cannot be employed with traditional fabrication technologies such as bioactive fluids that cannot be exposed to either photolithography or etching chemicals [22]. The patterns are defined on the substrate by the ejection of the ink through the print-head without touching the substrate. The deposition from the print-head to the substrate is only influenced by the gravity force and air resistance.

In the case of fluids, drying of the printed layer before its use is required. During the drying process, the solvents are evaporated by chemical changes or crystallized. Furthermore, a post-treatment is normally needed to make the deposited layer fully operative, such as thermal annealing or photonic sintering, [24,26] but without any particular processing conditions.

Inkjet printing can be applied to almost any substrate, from rigid to flexible ones, and from smooth to rough surfaces [19,27]. Some common employed substrates are glass [28], plastic [27,29], paper [30,31], and textile [32,33] among others.

It is also a fabrication technique which implies low consumption of raw materials [27] and environment-friendly production with low levels of waste that is harmful to the environment [19,34]. Many different applications have been developed by inkjet printing in recent years, such as in the production of transducers [22,35], transistors [17,36], structural polymers and ceramics [20], biomimetic and biomedical materials [21], printed scaffolds for the growth of living tissues [20], as well as for building 3D electric circuits [37], microelectromechanical systems (MEMS) [38,39], and sensors [23,40].

The inks utilized for inkjet printing have to fulfil some physical specifications related to superficial tension and viscosity level [27], and the ratio of humectant [41]. Also, it is crucial to select a suitable size of the particles of the ink to avoid the clogging of the nozzles. The drawbacks of this technique with compared to others is the low speed of the process, the thickness around nanometer range with one printed layer and the blocking of nozzles while printing because of clogging or drying of solvents. These disadvantages currently limit the use of inkjet printing at large scale. However, it is a perfect candidate to develop prototypes with high resolution and cost-effective manufacturing.

#### 2.1.2. Slot-Die

In the slot-die process, a thin film of solution, typically in the range of 5 to 30 µm, is applied homogenously through a slot die to the substrate surface. This technology offers high yield of manufacturing and high uniformity of the obtained films. It is commonly used in commercial roll-to-roll fabrication. One limitation of slot-die is the conductivity of the defined films because of the remaining surfactant molecules after deposition [42]. The other main limitation is the difficulty to define patterns, which reduces their use in complex structures and applications.

#### 2.1.3. Spray Deposition

Spray deposition is inherently a coating technique, which can easily be adapted to define patterns by using a shadow mask [43]. Spray deposition consists of the definition of a material, normally, in liquid form through a gas stream. The bulk material is divided into droplets, which is commonly known as an atomization process, to be subsequently carried to the substrate in a mixture of gas and a stream of droplets. There are two ways to achieve atomization by mixing with an air stream (air-assisted atomization) or by kinetic energy (ultrasonic atomization) [44]. In the case of air-assisted nozzles, a high velocity gas stream is required to generate atomization. The material can be fed into the nozzle under pressure or gravity. At the opening, the material to be deposited is mixed with the gas stream, forcing atomization. Whereas, in the case of ultrasonic-assisted nozzles, a piezoelectric transducer vibrating at ultrasonic frequencies is employed. When this vibrating element is put in contact with the material to be deposited, it becomes unstable, producing the droplets. Normally, the droplets are mixed with a carrier gas to be transported from the nozzle to the surface. In both types of atomization, the droplet size depends on many factors, such as nozzle dimensions, gas stream speed (in air-assisted atomization), or applied frequency (in ultrasonic-assisted atomization).

There are many examples in the literature where spray deposition is used for a large variety of materials, from thin-film transistors [45], organic photodiodes [46,47], organic photovoltaics [48], as well as chemical and biosensors [49,50] among others.

#### 2.1.4. Laser Direct Writing

The techniques based on laser direct-writing make possible the fabrication of 1D to 3D structures by laser induction of materials without any physical contact between the tool and the substrate material. The laser pulses, power and width, are controlled to obtain the expected composition structures and 3D properties across length-scales within the nanometers to millimeters spam [51]. We can differentiate between three types of laser direct writing techniques:

- Laser direct-writing addition technique, where the material can be deposited by transfer, with a laser beam, from an optically transparent support (e.g., laser-induced forward transfer [52]) or from gaseous, liquid and solid precursors (e.g., laser chemical vapor deposition [53]) onto the substrate. The equipment required for such technologies is sophisticated and of high cost; it is only possible to define materials on flat substrates parallel to the support materials and it does not allow printing on organic substrates.
- Laser direct-writing subtraction method, where the material is photo-removed (e.g., laser scribing [54], photochemical, photothermal, or photophysical ablation [55,56], cutting, drilling, or etching [57]). These techniques offer high-resolution manufacturing as well as the deposition of biomaterials.
- Laser direct-writing modification process, where the material is modified by a chemical solution or high local temperatures (e.g., laser-enhanced electroless plating) [51]. In particular, the substrate is submerged in a chemical solution with metallic ions needed for the deposition. Then, a laser beam rinses locally the temperature, decomposing the solution and, therefore, depositing a metallic layer on the surface. Its main limitation is the incapability to create 3D patterns.

#### 2.1.5. Aerosol Jet

Aerosol jet printing refers to the material deposition technique [58,59] developed by Optomec [60]. In this case, the solutions based on nanoparticles suspensions of different nature (from metals to polymers, adhesives or biomaterials) are placed into an atomizer where it aerosolizes in liquid particles of 20 nm to 5 μm diameter, depending on the solution viscosity. The transportation into the deposition head is carried out by a nitrogen flow, and then the aerosol focused by jet stream is deposited on the substrate. It is a low-temperature process, which facilitates the use of a wide range of materials and substrates. Thanks to the possibility of controlling the position of the z-axis of the printing head, 3D printed electronics can be developed with aerosol jet printing. It can be also scalable to mass production and complex designs (e.g., displays, thin film transistors, and solar cells) [60]. Its main drawbacks are the cloud of powder that is formed in the surroundings of the printing area, as well as the local solidification phase at the trace pattern provoked by the sheath gas, which reduces the bonding layer quality.

### 2.2. Contact Technologies

Contrary to the non-contact techniques, this group refers to those methods where a part of the printing equipment is also in contact with the substrate. This bunch of techniques implies high material volume wastes and they normally achieve poorer resolutions; however, they are the most predominant ones because of their high-speed production. In the following sections, the main contact printing technologies are described [16].

#### 2.2.1. Screen Printing

Screen printing can be applied in a roll-to-roll or a planar system. The planar process consists of using a screen mesh in direct contact with the substrate, a blade, which is in charge of distributing the paste and filling the mesh. The paste goes through the standard image defined in the mesh to the substrates by a squeegee, defining the final pattern. Many different substrates can be employed, such as epidermis [61], paper [62], glass [63], polymers [64], ceramic [65], and textiles [66]. Contrary to this, in the roll-to-roll screen printing technology, the squeegee is substituted by a roller and the blade and the paste go inside the roller. In this case, the fabrication is continuous, increasing the speed production [67]; however, these systems are more expensive and difficult to clean.

Screen printing is commonly used for PE [68,69]. Their main limitations are the waste of materials (screen, pastes, and cleaning solutions) and their poorer resolutions with respect to other printing technologies. However, it is quite easy to produce thick patterns in shorter times, which is particularly interesting in some applications [70].

#### 2.2.2. Flexography

Flexography is a roll-to-roll direct printing technique by ink transfer. It consists of a ceramic anilox roller with microcavities on its surfaces for the collection of the ink. The ink is supplied to the anilox roller by a closed chamber and it is then transferred to the printing plate cylinder, which continuously rotates in contact with the surface substrate [67]. The excess of ink is removed by a doctor blade, avoiding the output from the ink supply chamber.

Despite the high-speed and low-pressure process, there are limitations on image size and resolution because there are patterns with excess of ink due to the compression between the substrate and the printing plate roller (Halo effect) [67]. There are many examples in the literature where flexography technology is employed for piezoelectric pressure sensors [71], drug delivery patches, printed batteries, and smart radiofrequency identification tags [72,73].

#### 2.2.3. Gravure

Gravure is the reverse process of flexography, where the printed image is the negative. The ink arrives to the gravure plate where the pattern image is defined by the ink supplier container or by an additional roller. The transfer of the ink to the substrate happens through capillary from the small holes in the gravure cylinder. The excess of ink is removed by a flexible metal blade.

Gravure printing produces high quality patterns with high speed of fabrication (up to 0.1 m/s [74]). It is suitable for low viscosity inks. The final print quality depends on both the process parameters (i.e., cell spacing, feature dimensions on gravure cylinder, shear force in the printing mechanism) and the ink properties (i.e., its rheological behavior (viscosity), solvent evaporation rate, and curing) [75]. There are two main limitations of this technique:

- When printing straight lines, a jagged line is observed [67] because the printing image is built from separate cells. This is a handicap for high resolution patterns (less than 20 μm size is required for electronic structures [74])
- When printing consecutive layers (i.e., for conductive films to reduce sheet resistance), a proper alignment is required
- Also, when gravure is performed in roll-to-roll, it also has the extra inconvenience of frequent replacements of the gravure cylinders, implying higher maintenance cost

Gravure technology is utilized for some electronic products such as high-volume radio-frequency identification devices [76], thin-film-transistors [75], solar cells [77], and sensors [78].

#### 2.2.4. Soft Lithography

Soft lithography technology refers to several printing techniques, such as microcontact printing, replica molding, micro transference molding, micromolding in capillaries, and solvent assisted micromolding [79,80,81]. It provides high-quality micro and nanostructure systems [82].

This group of technologies utilizes an elastomeric stamp (normally of poly(dimethylsiloxane) (PDMS)) or mold with patterned structures on its surface to transfer the patterns within the range from 30 nm to 100 μm [81].

To prepare the stamps a master is normally prepared using e-beam or photolithography, from which several stamps can be molded. The material to be deposited is put on the stamp and transferred onto the substrate. However, its throughput is lower than roll-to-roll techniques [83] and the manufacturing involves several steps, including photolithographic technology [17,84]. There is also the limitation on the proper adjustment of the surface energies of inks and substrates to achieve an efficient transfer to the substrate, leading normally to a swelling of transferring materials, which increases the features size.

### 2.3. Printing Techniques for the Definition of Capacitive Structures

Although the conventional structure for capacitors is the PP and they are also developed with printed technologies, a very popular solution for printed sensors is planar capacitance where both electrodes are located in the same plane [85]. This kind of planar capacitance allows for contacting the device from only one side, leaving the other plane exposed to the ambient environment. Those sensors where a deformation of the dielectric is required, such as force sensors based on silicone [86,87], are normally used as PP. However in the case of sensors where the sensing mechanism is changes in the electrical permittivity, the utilization of planar electrodes is preferable due to its ease of fabrication [88,89].

With respect to the technology, when thin electrodes are needed, inkjet printing is preferable because the average thickness is hundreds of nanometers. Also, it is possible with this technique to obtain a high spatial resolution, typically around 20–50 µm. However, some materials commonly used materials for capacitive sensors, such as PDMS, are not compatible with this technique because of their high viscosity. Actually, an alternative is to combine different techniques to optimize the sensor performance [90,91].

## 3. Physical Capacitive Sensors

As commented in the introduction, these are physical sensors that quantify variations in physical properties of the devices under test (DUT), including axial and shear forces, acceleration, temperature, density, stiffness, thickness, bending stiffness, elastic modulus, presence of irregularities (cracks, voids, and edges), defects, among others [92]. In this section, we give an overview of the main physical capacitive sensors manufactured by PE in the last years.

### 3.1. Force and Pressure Sensors

Capacitive sensors have been widely employed to detect normal and shear forces as well as strain [93,94,95] because they can be manufactured with small dimensions, giving high spatial resolution. In fact, an array of different capacitive sensors can be used to detect and discriminate among forces in all directions [96]. Several groups have deployed tactile sensor arrays [97,98].

**Figure 2:**
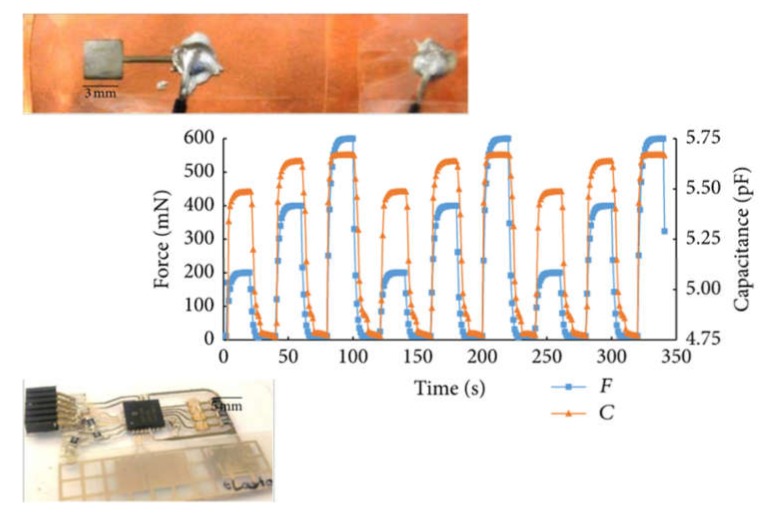
Dynamic response of the force sensor with 100 μm polydimethylsiloxane (PDMS) thick from [86]. Insets: Upside—Picture of the fabricated sensor. Downside—Hybrid integrated system with inkjet-printed silver layers and PDMS force sensor.

**Figure 3:**
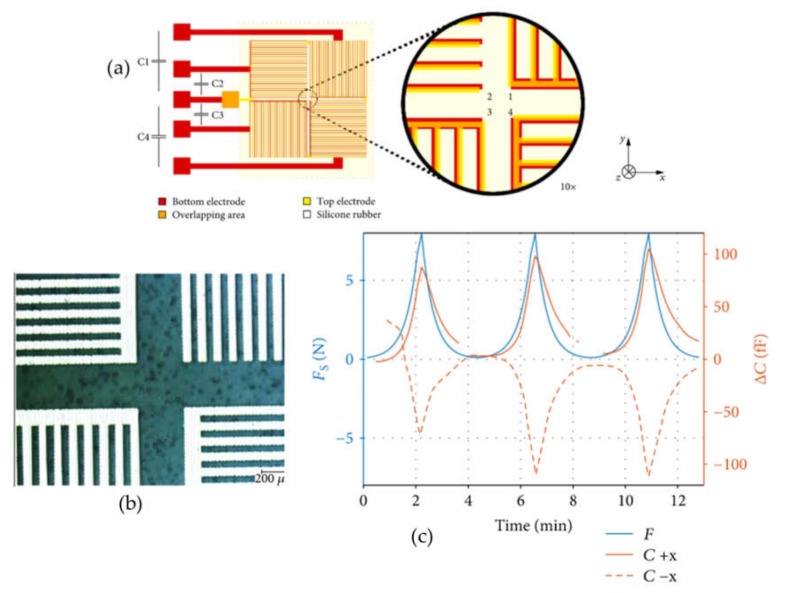
(**a**) Design of printed shear force sensor from [99]; (**b**) Image of the inkjet silver electrodes; (**c**) Capacitance response (red) to a normal force (blue) over time. (**b**) The solid red line corresponds to the capacitance change due to a force in – direction and the dashed line to the + direction.

A common material employed for the development of force sensors is PDMS. El-Mola et al. designed a simple PP capacitor with PDMS as dielectric and studied the influence of its thickness in the capacitive force sensor response [86] (see Figure 2). The PDMS films were placed on a polyimide foil with copper layer, acting as bottom electrode, and the top electrode was inkjet printed on the other side of the spin-coated PDMS film. The sensor with a 45-μm PDMS thick exhibited a sensitivity of about 3 pF/N which corresponded to a 45% change in capacitance. However, when the PDMS thickness was increased, the sensor sensitivity to applied normal force was significantly decreased. Although thinner PDMS films provided higher sensitivities, their dynamic response suffered, requiring more time to recover their initial capacitance. These authors showed that the best compromise between sensitivity and dynamic response was achieved with a PDMS film of 100 μm thickness. For such film, the sensor presented a nonlinear sensitivity of about 1.1 pF/N and a recovery time below 15 s without a noticeable hysteresis. Moreover, they integrated such a sensor with a microcontroller on a polyethylene terephthalate (PET) substrate, defining the interconnects with inkjet printing of silver nanoparticles, demonstrating the suitability of this technology for artificial skin applications. Using similar materials and techniques, Albrecth et al. described a capacitive sensor capable of detecting two-axial forces (see Figure 3). In particular, they defined structured photonic sintered silver electrodes on PET foil by inkjet printing with lines of 60 μm width and 500 nm thick. To build the sensor, two PET sheets printed with this structured electrode were glued with with a PDMS spacer in between. As the objective was to detect normal and shear forces in the same area, a 2 × 2 arrangement of four sensors was set to detect simultaneously normal and shear forces (three-axial). Although the experimental capacitances were lower than the theoretical predictions, a linear relationship in the 0.1 to 8 N force range was found with a sensitivity of 5.2 fF/N for normal forces and of 13.1 fF/N for shear forces. These values correspond to only one quarter of the sensor (about 80 mm^2^). When the array of sensors was characterized, displaying each of the array element in a different orientation, the shear force sensitivity to normal forces is quadrupled while the one to shear forces is doubled. The performances were comparable to previous sensors in the literature with the peculiarity that only additive deposition techniques with cost-effective materials were employed in this work [99].

Another example of a printed capacitive pressure sensor on a flexible substrate was described by Joo et al. [100]. They defined the electrodes with inkjet printed silver and spin coated on top a poly(methyl methacrylate) (PMMA) solution as dielectric layer. The top electrode was laminated on top of the dielectric layer with a silver nanowire (AgNW) embedded multiscale-structured PDMS. The sensor presented a non-linear sensitivity with 3.8 kPa^−1^ from 45–500 Pa, 0.8 kPa^−1^ from 500 Pa–2.5 kPa and 0.35 kPa^−1^ from 2.5–4.5 kPa, being capable of detecting very small pressure values (about 15 Pa). The time response and recovery are below 150 ms. They also demonstrated the scalability of the manufacturing process as well as the possibility of detecting the spatial distribution of the applied pressure with an array of such sensors. One year later, the same research group proposed another flexible pressure sensor with a tunable sensitivity [101]. In this case, they sprayed AgNWs on a buckled mold of PDMS with three different formation ratios of the PDMS crosslinker to form a nanocomposite. Again, the bottom electrode was defined by inkjet printing of silver nanoparticles on a poly(ethylene2, 6-naphtharate) (PEN) substrate. They confirmed that the shape dependence of the buckled structure on the PDMS mixing ratio, giving the greatest relative change for the nanowire composite with a 10:1 mixing ratio. Thanks to the mechanical differences in the PDMS matrix because of the mixing ratio and the shape of the crest area of the buckled structure, the pressure sensitivity can be easily tuned. Furthermore, the described pressure sensor can also detect the bending strain with a very stable response over repeated bending cycles.

Woo et al. presented skin-like sensors manufactured by a combination of soft-lithographic replication and contact printing based micro-patterning of a conductive elastomeric ink carbon nanotube (CNT)-doped PDMS. The array of capacitive sensors showed mechanical robustness against stretching, bending, twisting, and folding deformations without inducing any mechanical alteration in the sensor structure (such as cracks or exfoliation). The electrical responses of the array were highly linear and with very low hysteresis. Moreover, the individual responses of each capacitive cell of the array showed a relatively small deviation in both initial and deformed states, feature desirable for detecting spatial tactile information. They also demonstrated the practical application of their devices to artificial skins, detecting different types of human motions and spatial pressure distributions generated by different protrusion patterns [102]. Another example, where a carbon-based silicone composite was used as dielectric for a capacitive force sensor, was presented by Guo et al. [103]. They fabricated a capacitive pressure sensor based on a dielectric made of carbon black and silicone rubber. An organo-silicone conductive silver adhesive was used for the electrodes and the fabrication technique was screen-printing. The sensors exhibited a sensitivity of about 0.025%/KPa, a hysteresis error below 6%, and a dynamic response time of ~90 ms in the pressure range from 0 to 700 kPa. They also studied and modeled the influence of temperature in the sensor response, providing a calibration with thermal compensation. They demonstrated the applicability of the designed device to plantar pressure measurements as well as a soft-grasping manipulator, resulting in a promising candidate for wearable artificial skin.

Other common capacitive pressure sensors are capacitive acoustic transducers. An example of a printed one was described by Haque et al. [104]. They combined 3D printing and 2D inkjet printing to produce the devices. The printing of conductive layers on 3D printed substrates was optimized to get a proper conductivity without damaging the 3D structures. The membrane was fabricated on a pre-tensed film using thin organic films. The capacitive transducers exhibited selective sensitivity at their resonance frequency.

### 3.2. Accelerometers

Another type of physical sensors are accelerometers, which are able to measure the acceleration of objects in motion along reference axes. They are widely used in automotive applications, from the activation of safety systems to the electronic suspension and stability of the vehicle. Accelerometers are also extensively used in biomedical applications for activity monitoring [105,106].

In the case of printed capacitive sensors for acceleration, Ando et al. developed an inkjet printed accelerometer in the low frequency domain typical of inertial stimuli coming from human causes and seismic phenomena. Such accelerometer consisted of a PET membrane 200 µm thick clamped by four spring-legs to a fixed support. Four inkjet printed strain gauges made of silver were manufactured connected in series to readout the acceleration. A proof mass was placed in the center of the sensor area to increase the sensitivity and, at the same time, limit the device bandwidth [107]. The sensor response was 22 mV/g at 20 Hz and 42 mV/g at 45 Hz. Despite the interesting characteristics of this prototype, they redesigned the layout to enhance the performance of the accelerometer whose response was penalized by the rigid architecture sustaining the suspended plate [108]. This new accelerometer works up to 20 Hz with a flat response, which is the typical range in the field of seismic and human activity monitoring, thanks to the realization of both the membrane and the supporting springs by the flexible PET substrate. The sensor sensitivity and resolution at different frequencies were 9.4 mV/g and 0.126 g, respectively, at 10 Hz, and 41.0 mV/g and 0.003 g, respectively, at 35 Hz.

A common structure employed in the development of accelerometers are cantilever beams [109]. Such structure consists of a suspended element, which is not trivial to obtain with printing technologies. A fabrication method to facilitate the realization of printed cantilevers of flexible substrates wase described by Rivadeneyra et al. [110]. In this case, instead of using a sacrificial layer, they followed a process similar to wafer bonding in semiconductor device technology, utilizing a flipped sacrificial substrate. This sacrificial substrate was placed on top of the suspended structure instead of inserting this layer in the middle of it, as with conventional sacrificial layers. In particular, they succeeded in the manufacturing of a silver cantilever beam using PMMA as sacrificial substrate, which was removed at the end of the manufacturing process by an acetone bath. The cantilever deflections at different values of acceleration and frequency oscillation were investigated to prove the reliability of the proposed fabrication method. Also, the change in capacitance while the cantilever was shaking was also measured at some values of acceleration, showing their potential for the development of printed capacitive accelerometers on flexible substrates. An improvement in this fabrication process was described in [111]. In this case, poly(vinyl alcohol) (PVA) was employed as sacrificial substrate, who can be dissolved in water and it does not react with the silver layer contrary to the acetone bath required for PMMA. This second approach increased the yield rate and the fabricated cantilevers were flatter than the previous ones. Their responses in terms of displacement and capacitance at different accelerations and frequencies were also presented, showing similar behavior to the previous one. It was also demonstrated that the thickness of the cantilever pillar can be controlled by the number of printed layers.

### 3.3. Strain Sensors

The last type of physical capacitive sensors fabricated by printing techniques described in this document are strain sensors, which are conventionally designed as resistive sensors but there are also examples of capacitive ones. An example of a printed capacitive device for strain detection was reported by Cholleti et al. [112]. In particular, they defined an interdigitated electrode made of a carbon black rubber sandwiched between a barium titanate (BTO) elastomer composite stretchable substrate. The first finding was the increase in the nominal capacitance by adding 200 nm of BTO to the elastomer, facilitating the detection of the sensor capacitance. The addition of 40 wt% BTO resulted in a 44% change in capacitance when stretched 100% with high repeatability over 100 cycles.

Vazquez-Quintero et al. described an inkjet printed sensor fabricated on cylindrical PET fibers used in industrial textiles [33]. The capacitance change showed a maximum value of 0.7% at 1% of strain. The functionalized fibers were woven with metallic ones, which resulted interesting for automotive industry and predictive maintenance of industrial textile.

Yae et al. developed a highly stretchable multifunctional capacitive sensor capable of detecting strain (up to 50%), pressure (up to ~1.2 MPa) and finger touch with a high sensitivity and a fast response [113]. Such a sensor was fabricated by screen printing silver nanowire electrodes on Ecoflex substrate, acting as dielectric. They tested the sensors for several wearable applications such as thumb movement monitoring, strain sensing of the knee joint in patellar reflex among other human motions. The sensor exhibited a quite linear response to large tensile strain up to 50% with a gauge factor of ~0.7 and a bilinear response to pressure with capacitance values of few pF.

In the work of Žlebič et al. [102], they compared resistive and capacitive strain gauge sensors fabricated by inkjet printing based on silver nanoparticles electrodes on polyimide substrate.

## 4. Chemical Capacitive Sensors

Chemical sensors cover a wide range of devices that can potentially monitor electrolytes [114,115], metabolites [116,117], heavy metals [118,119], and gases [120,121]. In this section, we summarize the main contributions in the field of printed chemical capacitive sensors.

### 4.1. Relative Humidity Detectors

**Figure 4:**
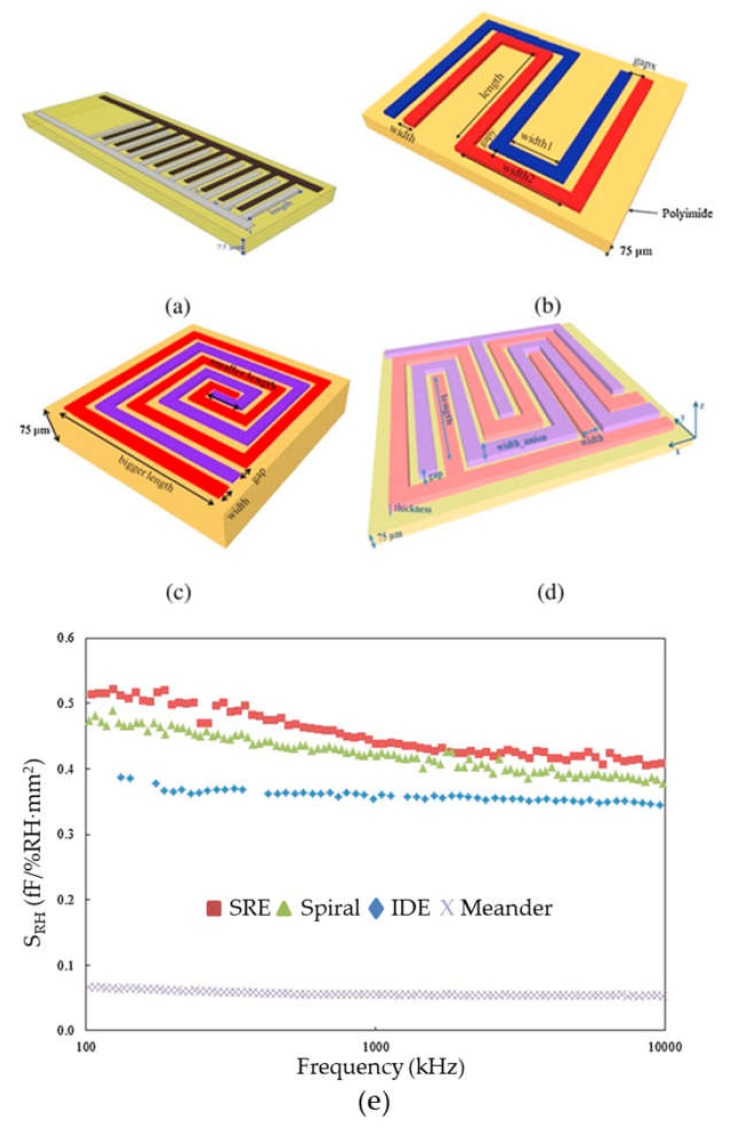
Layout of the designed and characterized electrodes in [85]: (**a**) interdigitated electrodes (IDE) sensor; (**b**) Meandered capacitor; (**c**) Spiral electrodes and (**d**) Serpentine electrode (SRE) capacitor. (**e**) Normalized sensitivity to RH as a function of frequency for each electrode configuration.

In the case of chemical capacitive sensors, relative humidity (RH) is the most common one in the literature. Gaspar et al. described an inkjet printed sensor made defining silver interdigitated electrodes on a paper substrate. In fact, the sensitive layer is directly the substrate. The sensor exhibited a sensitivity of about 2 pF/%RH with a relative fast response and without showing significant thermal drift in the range from about 5–85 °C [122]. Using the same approach of having a sensitive substrate as sensing layer, Rivadeneyra et al. presented a comparison among different planar layouts of silver electrodes inkjet printed on a polyimide foil [85]. In particular, the four tested electrodes were the following: interdigitated electrodes, meandered electrodes, spiral electrodes, and serpentine electrodes. They proved the feasibility of developing capacitive RH sensors, highlighting the advantages and drawbacks of each geometry. For example, the highest sensitivity to RH was found for serpentine electrodes, followed by spiral ones, interdigitated electrodes (see Figure 4). The lowest sensitivity was obtained for meandered ones. In terms of thermal drift, spiral electrodes exhibited higher values than the other three geometries, whose sensitivity to temperatures was around ±0.5 fF mm^−2^/°C. This difference in the spiral electrodes was associated to the anisotropic temperature behavior of the employed polyimide, which was put in evidence only for those electrodes because the other electrode geometries were symmetric. The main conclusion of this work was the fact that no layout is better than the others; it is dependent on the final application. For instance, when the limiting factor is the RH sensitivity, spiral and serpentine electrodes should be selected. However, because of their more complex geometry, while printing, their yield rate is lower than the other two analyzed layouts. The best compromise between sensitivity to RH and yield rate is found for interdigitated electrodes while meandered ones facilitate their inclusion in more complex designs (such as the ones described here [91]).

A different technique for the fabrication of capacitive sensors for RH detection was followed by Christos et al. [123]. They used a combination of a laser engraving system to define interdigitated electrodes on PET substrate and spin coating for the deposition of the sensitive layer. In particular, the electrodes were done with gold while the sensitive material was Nafion. The non-linear sensitivity of the reported sensors in the range from 15%–95%RH was 12.27 fF/%RH and 40.86 fF/%RH, for RH < 65% and RH > 65% respectively. Another sensitive layer used for the development of capacitive RH sensors is a graphene (G)/methyl-red (M-R) composite [124]. In this sensor, silver interdigitated electrodes were inkjet printed on PET and the sensitive composite was deposited over then by electrohydrodynamics. The fabricated sensors presented a variation in capacitance from 2.3 pF to 66 nF in the range from 5%–95% RH with a fast response.

The work of Romero et al. described a comparison of interdigitated electrodes on flexible substrates fabricated with two different techniques and three different sensitive layers to RH [125]. Two of the capacitive sensors were fabricated by laser writing (laser scribing of reduced graphene oxide and laser induced graphene), while the other one was defined by inkjet printing of silver nanoparticles. This work highlighted the advantages and disadvantages of each technique for the manufacturing of RH sensors. For example, the laser induced graphene sensors showed a more linear response and the lowest thermal drift, whereas the inkjetted one exhibited lower losses and higher sensitivity to RH.

There are also examples of RH sensors fabricated by screen printing. For instance, a novel geometry based on interdigitated electrodes but defined out-of-plane in z-axis was described in [126]. What they did was to place each electrode at different heights. To further increase the sensitive surface, the bottom electrode was also connected to a planar plate. The sensitive layer was cellulose acetate butyrate. The sensor presented a high sensitivity to RH (about 5 pF/%RH at 1 MHz for a capacitance at normal conditions in the range of hundreds of pF). Also, Molina-Lopez et al. used the same sensitive layer but using inkjet printing for its deposition [29].

### 4.2. Gas and Vapour Sensing

There are a few examples of printed capacitive gas sensors. One of them was described by Bahoumina et al. [127]. They inkjet printed a capacitive microwave sensor on paper with a carbon composite polymer as sensitive material. The sensor was characterized against ethanol vapor, showing a sensitivity of about −2.5 kHz/ppm from 0 to 2000 ppm.

A similar approach was described in [128]. A capacitive microwave sensor made by inkjet printing, where the conductive patterns were defined with silver nanoparticles and the sensitive layer was a mixture of poly(3,4-ethylenedioxythiophene) polystyrene sulfonate (PEDOT:PSS) and multiwalled CNTs (MWCNTs). They studied the influence of the number of printed layer of the sensitive material in the sensor sensitivity, which is directly related to the thickness of the defined layer. In particular, they found a sensitivity to ethanol of 0.9 kHz/ppm and 1.3 kHz/ppm for 5 and 50 sensitive layers, respectively.

Another example of printed capacitive sensor for gas detection is described in the work of Kukkola et al. [129]. In particular, they combined inkjet printing and gravure printing for the development of three different gas sensors for NO and H_2_ detection. They were able to detect concentrations below 10 ppm and 100 ppm, respectively. The devices were manufactured on a plastic foil substrate.

## 5. Conclusions and Future Perspectives

We have analysed in this paper the different fabrication technologies available for manufacturing printed capacitive sensors, from contact (i.e., flexography and gravure printing) to non-contact techniques (i.e., inkjet printing and aerosol jet printing), highlighting their main characteristics. We have seen the utilization of such techniques for the development of many different types of sensor, differing in terms of their detection of physical and chemical magnitudes. We have seen through this paper that most of these sensors are produced on flexible and thin-film substrates, which is an extra feature offered by printed electronics, difficult to achieve with conventional fabrication techniques. Also, it is worth mentioning the employment of novel materials, such as graphene derivates, carbon nanotubes, and metallic nanowires, which provide outstanding properties in terms of biodegradability, stretchability, or biocompatibility.

In general terms, the mostly utilized techniques are screen printing and inkjet printing. The former is widely used because of the ease of manufacturing while the later provides patterns with high resolution and wastes less materials, resulting very attractive for prototyping purposes. However, other techniques are becoming also very popular. In particular, laser writing is being used for inducing graphene derivates and spray deposition to define thin sensing layers. It is worth mentioning that, in recent years, the combination of printing techniques to optimize the size and performance of capacitive structures has become popular.

The most advanced printed capacitive sensors include pressure and force sensors, temperature sensors and relative humidity detectors. There are also examples of printed accelerometers and gas sensors, but they are more limited than the other examples because of the difficulty of defining suspended structures by printed techniques (for accelerometers) and the need of investigating the selectivity of dielectrics for gas detection and the need of investigating the selectivity of dielectrics for gas detection.

In conclusion, many efforts have been made to develop fully printed sensor solutions, and many research groups are dedicated to this research topic worldwide. This large amount of work is motivated by the promise of achieving complete systems that can be printed on mechanically flexible substrates, with many desirable properties such as low cost, environmental sustainability, wide variety of substrates, print on demand, and scalable technologies. Such systems are increasingly recognized as a key element in the Internet of Things (IoT), as part of the “Fourth Industrial Revolution”.

However, a number of issues related to very high-volume production still need to be overcome. Firstly, the manufacturing technique has to be improved in order to be applied efficiently in the production of large-area, high-volume systems. For this mass production, some issues that are usually well-controlled in the production of small prototypes in the laboratory have to be extended to the industrial environment. The main current limitations of printed electronics to develop capacitive sensors are the roughness of the printed layers when stacking is needed together with the thickness and resolution of the patterns to increase the performance of the capacitive device. Other examples of such issues are device testing and calibration, resolution, accuracy of overlapping layers when printing on top of each other, uniformity and variations, and throughput. Secondly, sensors must be integrated with electronic printed circuits for information processing and communication. Sensor technology must be accompanied by the development of suitable printed transistors, which pose additional challenges in terms of both materials and design, such as increased mobility of the charge carriers, complementary devices, solution processability, environmental stability and low operating voltage. For systems requiring intensive data processing, compatibility with silicon devices that can be encapsulated on flexible substrates is also an interesting issue. Circuit design methodologies for silicon, based on relatively low variability, may not be directly applicable and would require co-design and cooperation between the different components. Finally, three-dimensional structures can also be envisaged. Sensors of different types, or other devices such as antennas, may be stacked, and printed electronics can offer this possibility, both by stacking 2D layers and by integration with 3D printing. Although 3D printing seems not to have taken off as expected, much progress can be made in this direction with the addition of new materials.

In short, as has happened with other emerging technologies in the past, we can foresee the emergence of intelligent, large-area printed systems, which could even open up new applications in the near future.

## Figures and Tables

**Table 1:** Sensor classifications.

Classification	Types	Definition/ Method*
**Energy contribution**	Modulator/Passive	An external source of power is needed to provide the majority of the output power of the signal
	Generator/Active	The output power is virtually provided by the measured signal (no excitation voltage is required). They produce an output signal in the form of some variation in resistance, capacitance or any other electrical parameter, which then has to be converted to an equivalent current or voltage signal
**Output signal**	Analog	Analog sensors produce a continuous signal in relation with the measurand signal
	Digital	Digital sensors provide a binary signal
**Nature of the information**	Chemical	A chemical sensor transforms chemical information into an analytically useful signal, such as gas and ion concentration
	Physical	A physical sensor gives information about a physical property of the system, such as temperature, density or speed
	Biological	A biosensor (biological sensor) combines a biological component with a physicochemical detector
Transduction Mechanism*	Mechanical	Stress sensing, Mass sensing
	Optical	Fluorescence, Chemilumiscence, Bioluminescence, Surface Plasmon, Scattering, Evanescent Waves Interferometry
	Electrical	Conductometric, Capacitive
	Piezoelectric	Quarzt Crystal Microbalance, Surface Acoustic Wave
	Electrochemical	Potentiometric, Amperiometric, Ion Sensitive Field Effect Transistor (FET) Chemical FET
	Thermal	Calorimetric

* Method: Type of transduction mechanism.

## References

[1] Fraden J. (2004). Handbook of Modern Sensors: Physics, Designs, and Applications.

[2] Wang P., Liu Q. (2011). Biomedical Sensors and Measurement.

[3] Bremner D. (2015). The Importance of Sensors to the Internet of Things. https://eprints.gla.ac.uk/105145/.

[4] Vetelino J., Reghu A. (2017). Introduction to Sensors.

[5] Sukhija M., Nagsarkar T. (2010). Circuits and Networks: Analysis, Design, and Synthesis.

[6] Baxter L.K. (1996). Capacitive Sensors: Design and Applications.

[7] Precision L. (2012). Capacitive Sensor Operation and Optimization.

[8] Eren H. (2014). Capacitive sensors. Measurement, Instrumentation, and Sensors Handbook: Spatial, Mechanical, Thermal, and Radiation Measurement.

[9] Huang S., Plaskowski A., Xie C., Beck M. (1989). Tomographic imaging of two-component flow using capacitance sensors. J. Phys. E Sci. Instrum..

[10] Xie C., Stott A., Plaskowski A., Beck M. (1990). Design of capacitance electrodes for concentration measurement of two-phase flow. Meas. Sci. Technol..

[11] Gaitán-Pitre J.E., Gasulla M., Pallàs-Areny R. Direct interface for capacitive sensors based on the charge transfer method. Proceedings of the 2007 IEEE Instrumentation & Measurement Technology Conference, IMTC 2007.

[12] Haider M., Mahfouz M., Islam S., Eliza S., Qu W., Pritchard E. A low-power capacitance measurement circuit with high resolution and high degree of linearity. Proceedings of the 2008 IEEE International 51st Midwest Symposium on Circuits and Systems.

[13] Segundo A.K.R., Martins J.H., Monteiro P.M.d.B., Oliveira R.A.d., Oliveira Filho D. (2011). Development of capacitive sensor for measuring soil water content. Eng. Agríc..

[14] Perelaer J., Smith P.J., Mager D., Soltman D., Volkman S.K., Subramanian V., Korvink J.G., Schubert U.S. (2010). Printed electronics: The challenges involved in printing devices, interconnects, and contacts based on inorganic materials. J. Mater. Chem..

[15] Suganuma K. (2014). Introduction to Printed Electronics.

[16] Cruz S.M.F., Rocha L.A., Viana J.C. (2018). Printing technologies on flexible substrates for printed electronics. Flexible Electronics.

[17] Kawase T., Shimoda T., Newsome C., Sirringhaus H., Friend R.H. (2003). Inkjet printing of polymer thin film transistors. Thin Solid Films.

[18] Cui Z. (2016). Printed Electronics: Materials, Technologies and Applications.

[19] Nir M. (2010). Electrically Conductive Inks for Inkjet Printing the Chemistry of Inkjet Inks ed S Magdassi.

[20] Calvert P. (2001). Inkjet printing for materials and devices. Chem. Mater..

[21] Calvert P., Yoshioka Y., Jabbour G. (2004). Inkjet printing for biomimetic and biomedical materials. Learning from Nature How to Design New Implantable Biomaterialsis: From Biomineralization Fundamentals to Biomimetic Materials and Processing Routes.

[22] Al-Chami H. (2010). Inkjet Printing of Transducers.

[23] Rivadeneyra A., Bobinger M., Albrecht A., Becherer M., Lugli P., Falco A., Salmerón J.F. (2019). Cost-effective pedot: Pss temperature sensors inkjetted on a bendable substrate by a consumer printer. Polymers.

[24] Albrecht A., Rivadeneyra A., Abdellah A., Lugli P., Salmerón J.F. (2016). Inkjet printing and photonic sintering of silver and copper oxide nanoparticles for ultra-low-cost conductive patterns. J. Mater. Chem. C.

[25] Crowley K., Morrin A., Hernandez A., O’Malley E., Whitten P.G., Wallace G.G., Smyth M.R., Killard A.J. (2008). Fabrication of an ammonia gas sensor using inkjet-printed polyaniline nanoparticles. Talanta.

[26] Perelaer J., Schubert U.S., Jena F. (2010). Inkjet printing and alternative sintering of narrow conductive tracks on flexible substrates for plastic electronic applications. Radio Frequency Identification Fundamentals and Applications, Design Methods And Solutions.

[27] De Gans B.J., Duineveld P.C., Schubert U.S. (2004). Inkjet printing of polymers: State of the art and future developments. Adv. Mater..

[28] Abulikemu M., Da’As E.H., Haverinen H., Cha D., Malik M.A., Jabbour G.E. (2014). In situ synthesis of self-assembled gold nanoparticles on glass or silicon substrates through reactive inkjet printing. Angew. Chem..

[29] Molina-Lopez F., Briand D., de Rooij N. (2012). All additive inkjet printed humidity sensors on plastic substrate. Sens. Actuators B Chem..

[30] Andersson H., Manuilskiy A., Unander T., Lidenmark C., Forsberg S., Nilsson H. (2012). Inkjet printed silver nanoparticle humidity sensor with memory effect on paper. Sens. J. IEEE.

[31] Courbat J., Kim Y., Briand D., de Rooij N. Inkjet printing on paper for the realization of humidity and temperature sensors. Proceedings of the 2011 16th International Solid-State Sensors, Actuators and Microsystems Conference (TRANSDUCERS).

[32] Ujiie H. (2006). Digital Printing of Textiles.

[33] Quintero A.V., Camara M., Mattana G., Gaschler W., Chabrecek P., Briand D., de Rooij N. (2015). Capacitive strain sensors inkjet-printed on pet fibers for integration in industrial textile. Proced. Eng..

[34] Ando B., Baglio S. (2011). Inkjet-printed sensors: A useful approach for low cost, rapid prototyping [instrumentation notes]. IEEE Instrum. Meas. Mag..

[35] Andò B., Baglio S., Bulsara A.R., Emery T., Marletta V., Pistorio A. (2017). Low-cost inkjet printing technology for the rapid prototyping of transducers. Sensors.

[36] Okimoto H., Takenobu T., Yanagi K., Miyata Y., Shimotani H., Kataura H., Iwasa Y. (2010). Tunable carbon nanotube thin-film transistors produced exclusively via inkjet printing. Adv. Mater..

[37] Mei J., Lovell M.R., Mickle M.H. (2005). Formulation and processing of novel conductive solution inks in continuous inkjet printing of 3-d electric circuits. IEEE Trans. Electron. Packag. Manuf..

[38] Fuller S.B., Wilhelm E.J., Jacobson J.M. (2002). Ink-jet printed nanoparticle microelectromechanical systems. J. Microelectromech. Syst..

[39] Molina-Lopez F., Briand D., de Rooij N.F. Large arrays of inkjet-printed mems microbridges on foil. Proceedings of the IEEE 27th International Conference on Micro Electro Mechanical Systems (MEMS).

[40] Azzellino G., Grimoldi A., Binda M., Caironi M., Natali D., Sampietro M. (2013). Fully inkjet-printed organic photodetectors with high quantum yield. Adv. Mater..

[41] Caglar U. (2010). Studies of Inkjet Printing Technology with Focus on Electronic Materials.

[42] Hecht D., Grüner G. (2009). Solution cast films of carbon nanotubes for transparent conductors and thin film transistors. Flexible Electronics.

[43] Rivadeneyra A., Loghin F.C., Falco A. (2018). Technological integration in printed electronics. Flexible Electronics.

[44] Lefebvre A.H. (1989). Atomization and Sprays.

[45] Loghin F., Colasanti S., Weise A., Falco A., Abdelhalim A., Lugli P., Abdellah A. (2016). Scalable spray deposition process for highly uniform and reproducible cnt-tfts. Flex. Print. Electron..

[46] Abdellah A., Fabel B., Lugli P., Scarpa G. (2010). Spray deposition of organic semiconducting thin-films: Towards the fabrication of arbitrary shaped organic electronic devices. Org. Electron..

[47] Falco A., Cinà L., Scarpa G., Lugli P., Abdellah A. (2014). Fully-sprayed and flexible organic photodiodes with transparent carbon nanotube electrodes. ACS Appl. Mater. Interfaces.

[48] Steirer K.X., Reese M.O., Rupert B.L., Kopidakis N., Olson D.C., Collins R.T., Ginley D.S. (2009). Ultrasonic spray deposition for production of organic solar cells. Sol. Energy Mater. Sol. Cells.

[49] Abdelhalim A., Winkler M., Loghin F., Zeiser C., Lugli P., Abdellah A. (2015). Highly sensitive and selective carbon nanotube-based gas sensor arrays functionalized with different metallic nanoparticles. Sens. Actuators B Chem..

[50] Münzer A., Heimgreiter M., Melzer K., Weise A., Fabel B., Abdellah A., Lugli P., Scarpa G. (2013). Back-gated spray-deposited carbon nanotube thin film transistors operated in electrolytic solutions: An assessment towards future biosensing applications. J. Mater. Chem. B.

[51] Arnold C.B., Piqué A. (2007). Laser direct-write processing. MRS Bull..

[52] Araki T., Mandamparambil R., Jiu J., Sekitani T., Suganuma K. (2017). Application of printed silver nanowires based on laser-induced forward transfer. Nanomaterials for 2D and 3D Printing.

[53] Mazumder J., Kar A. (2013). Theory and Application of Laser Chemical Vapor Deposition.

[54] El-Kady M.F., Strong V., Dubin S., Kaner R.B. (2012). Laser scribing of high-performance and flexible graphene-based electrochemical capacitors. Science.

[55] Romero F., Salinas-Castillo A., Rivadeneyra A., Albrecht A., Godoy A., Morales D.P., Rodriguez N. (2018). In-depth study of laser ablation of kapton polyimide for flexible conductive substrates. Nanomaterials.

[56] Romero F.J., Rivadeneyra A., Toral V., Castillo E., García-Ruiz F., Morales D.P., Rodriguez N. (2018). Design guidelines of laser reduced graphene oxide conformal thermistor for iot applications. Sens. Actuators A Phys..

[57] Migliore L.R. (2018). Laser Materials Processing.

[58] Christenson K.K., Paulsen J.A., Renn M.J., McDonald K., Bourassa J. (2011). Direct printing of circuit boards using aerosol jet^®^. Proceedings of the NIP & Digital Fabrication Conference.

[59] Feng J.Q., Renn M.J. (2019). Aerosol jet^®^ direct-write for microscale additive manufacturing. J. Micro Nano-Manuf..

[60] Optomec Optomec. https://www.optomec.com/.

[61] Li M., Li Y.-T., Li D.-W., Long Y.-T. (2012). Recent developments and applications of screen-printed electrodes in environmental assays—A review. Anal. Chim. Acta.

[62] Yafia M., Shukla S., Najjaran H. (2015). Fabrication of digital microfluidic devices on flexible paper-based and rigid substrates via screen printing. J. Micromech. Microeng..

[63] Ito S., Chen P., Comte P., Nazeeruddin M.K., Liska P., Péchy P., Grätzel M. (2007). Fabrication of screen-printing pastes from tio2 powders for dye-sensitised solar cells. Prog. Photovolt. Res. Appl..

[64] Albrecht A., Salmeron J.F., Becherer M., Lugli P., Rivadeneyra A. (2019). Screen-printed chipless wireless temperature sensor. IEEE Sens. J..

[65] Metters J.P., Kadara R.O., Banks C.E. (2013). Fabrication of co-planar screen printed microband electrodes. Analyst.

[66] Locher I., Tröster G. (2007). Screen-printed textile transmission lines. Text. Res. J..

[67] Blayo A., Pineaux B. (2005). Printing processes and their potential for rfid printing. Proceedings of the 2005 Joint Conference on Smart Objects and Ambient Intelligence: Innovative Context-Aware Services: Usages and Technologies.

[68] Castellanos-Ramos J., Navas-González R., Macicior H., Sikora T., Ochoteco E., Vidal-Verdú F. (2010). Tactile sensors based on conductive polymers. Microsyst. Technol..

[69] Crowley K., Morrin A., Shepherd R.L., Wallace G.G., Smyth M.R., Killard A.J. (2010). Fabrication of polyaniline-based gas sensors using piezoelectric inkjet and screen printing for the detection of hydrogen sulfide. Sens. J. IEEE.

[70] Salmerón J.F., Torres A.R., Banqueri J., Carvajal M.A., Agudo M. Design and characterization of ink-jet and screen printed hf rfid antennas. Proceedings of the 2012 Fourth International EURASIP Workshop on RFID Technology (EURASIP RFID 2012).

[71] Julin T. (2012). Flexo-Printed Piezoelectric Pvdf Pressure Sensors. Master’s Thesis.

[72] Vena A., Perret E., Tedjini S., Tourtollet G.E.P., Delattre A., Garet F., Boutant Y. (2013). Design of chipless rfid tags printed on paper by flexography. IEEE Trans. Antennas Propag..

[73] Maddipatla D., Narakathu B.B., Avuthu S.G.R., Emamian S., Eshkeiti A., Chlaihawi A.A., Bazuin B.J., Joyce M.K., Barrett C.W., Atashbar M.Z. A novel flexographic printed strain gauge on paper platform. Proceedings of the 2015 IEEE Sensors.

[74] Clark D.A. (2010). Major Trends in Gravure Printed Electronics.

[75] Sung D., de la Fuente Vornbrock A., Subramanian V. (2009). Scaling and optimization of gravure-printed silver nanoparticle lines for printed electronics. IEEE Trans. Compon. Packag. Technol..

[76] Park H., Kang H., Lee Y., Park Y., Noh J., Cho G. (2012). Fully roll-to-roll gravure printed rectenna on plastic foils for wireless power transmission at 13.56 MHz. Nanotechnology.

[77] Yang J., Vak D., Clark N., Subbiah J., Wong W.W., Jones D.J., Watkins S.E., Wilson G. (2013). Organic photovoltaic modules fabricated by an industrial gravure printing proofer. Sol. Energy Mater. Sol. Cells.

[78] Lee W., Koo H., Sun J., Noh J., Kwon K.-S., Yeom C., Choi Y., Chen K., Javey A., Cho G. (2015). A fully roll-to-roll gravure-printed carbon nanotube-based active matrix for multi-touch sensors. Sci. Rep..

[79] Liu C.-X., Choi J.-W. (2009). Patterning conductive pdms nanocomposite in an elastomer using microcontact printing. J. Micromech. Microeng..

[80] Molina-Lopez F., Briand D., de Rooij N.F. (2015). Inkjet and microcontact printing of functional materials on foil for the fabrication of pixel-like capacitive vapor microsensors. Org. Electron..

[81] Xia Y., Whitesides G.M. (1998). Soft lithography. Annu. Rev. Mater. Sci..

[82] Qin D., Xia Y., Whitesides G.M. (2010). Soft lithography for micro-and nanoscale patterning. Nat. Protoc..

[83] Basiricò L. (2012). Inkjet Printing of Organic Transistor Devices.

[84] Cheng M.-Y., Lin C.-L., Yang Y.-J. Tactile and shear stress sensing array using capacitive mechanisms with floating electrodes. Proceedings of the 2010 IEEE 23rd International Conference on Micro Electro Mechanical Systems (MEMS).

[85] Rivadeneyra A., Fernández-Salmerón J., Agudo-Acemel M., López-Villanueva J.A., Capitan-Vallvey L.F., Palma A.J. (2016). Printed electrodes structures as capacitive humidity sensors: A comparison. Sens. Actuators A Phys..

[86] El-Molla S., Albrecht A., Cagatay E., Mittendorfer P., Cheng G., Lugli P., Salmerón J.F., Rivadeneyra A. (2016). Integration of a thin film pdms-based capacitive sensor for tactile sensing in an electronic skin. J. Sens..

[87] Sethumadhavan V., Saraf S., Choudhari A., Gaikwad R. Flexible capacitive based printed sensor using different dielectrics for real time applications. Proceedings of the 2017 International Conference on Trends in Electronics and Informatics (ICEI).

[88] Altenberend U., Molina-Lopez F., Oprea A., Briand D., Bârsan N., De Rooij N.F., Weimar U. (2013). Towards fully printed capacitive gas sensors on flexible pet substrates based on ag interdigitated transducers with increased stability. Sens. Actuators B Chem..

[89] Rivadeneyra A., Fernández-Salmerón J., Banqueri J., Lopez-Villanueva J.A., Capitan-Vallvey L.F., Palma A.J. (2014). A novel electrode structure compared with interdigitated electrodes as capacitive sensor. Sens. Actuators B Chem..

[90] Falco A., Loghin F.C., Becherer M., Lugli P., Salmerón J.F., Rivadeneyra A. (2019). Low-cost gas sensing: Dynamic self-compensation of humidity in cnt-based devices. ACS Sens..

[91] Rivadeneyra A., Fernández-Salmerón J., Agudo-Acemel M., López-Villanueva J.A., Capitán-Vallvey L.F., Palma A.J. (2016). Hybrid printed device for simultaneous vapors sensing. IEEE Sens. J..

[92] Zhang P. (2010). Advanced Industrial Control Technology.

[93] Kanao K., Harada S., Yamamoto Y., Honda W., Arie T., Akita S., Takei K. (2015). Highly selective flexible tactile strain and temperature sensors against substrate bending for an artificial skin. RSC Adv..

[94] Lipomi D.J., Vosgueritchian M., Tee B.C., Hellstrom S.L., Lee J.A., Fox C.H., Bao Z. (2011). Skin-like pressure and strain sensors based on transparent elastic films of carbon nanotubes. Nat. Nanotechnol..

[95] Narakathu B., Eshkeiti A., Reddy A., Rebros M., Rebrosova E., Joyce M., Bazuin B., Atashbar M. A novel fully printed and flexible capacitive pressure sensor. Proceedings of the 2012 IEEE Sensors.

[96] Harada S., Kanao K., Yamamoto Y., Arie T., Akita S., Takei K. (2014). Fully printed flexible fingerprint-like three-axis tactile and slip force and temperature sensors for artificial skin. ACS Nano.

[97] Schwartz G., Tee B.C.-K., Mei J., Appleton A.L., Kim D.H., Wang H., Bao Z. (2013). Flexible polymer transistors with high pressure sensitivity for application in electronic skin and health monitoring. Nat. Commun..

[98] Ying M., Bonifas A.P., Lu N., Su Y., Li R., Cheng H., Ameen A., Huang Y., Rogers J.A. (2012). Silicon nanomembranes for fingertip electronics. Nanotechnology.

[99] Albrecht A., Trautmann M., Becherer M., Lugli P., Rivadeneyra A. (2019). Shear-force sensors on flexible substrates using inkjet printing. J. Sens..

[100] Joo Y., Byun J., Seong N., Ha J., Kim H., Kim S., Kim T., Im H., Kim D., Hong Y. (2015). Silver nanowire-embedded pdms with a multiscale structure for a highly sensitive and robust flexible pressure sensor. Nanoscale.

[101] Joo Y., Yoon J., Hong Y. (2016). Elastomeric nanowire composite for flexible pressure sensors with tunable sensitivity. J. Inf. Disp..

[102] Woo S.-J., Kong J.-H., Kim D.-G., Kim J.-M. (2014). A thin all-elastomeric capacitive pressure sensor array based on micro-contact printed elastic conductors. J. Mater. Chem. C.

[103] Guo X., Huang Y., Cai X., Liu C., Liu P. (2016). Capacitive wearable tactile sensor based on smart textile substrate with carbon black/silicone rubber composite dielectric. Meas. Sci. Technol..

[104] Haque R.I., Ogam E., Loussert C., Benaben P., Boddaert X. (2015). Fabrication of capacitive acoustic resonators combining 3d printing and 2d inkjet printing techniques. Sensors.

[105] Yazdi N., Ayazi F., Najafi K. (1998). Micromachined inertial sensors. Proc. IEEE.

[106] Söderkvist J. (1994). Micromachined gyroscopes. Sens. Actuators A Phys..

[107] Andò B., Baglio S., Lombardo C., Marletta V., Pistorio A. An inkjet printed seismic sensor. Proceedings of the 2015 IEEE International Instrumentation and Measurement Technology Conference (I2MTC).

[108] Andò B., Baglio S., Lombardo C.O., Marletta V., Pistorio A. (2015). A low-cost accelerometer developed by inkjet printing technology. IEEE Trans. Instrum. Meas..

[109] Kovacs A., Vízváry Z. (2001). Structural parameter sensitivity analysis of cantilever-and bridge-type accelerometers. Sens. Actuators A Phys..

[110] Rivadeneyra A., Fernández-Salmerón J., Agudo-Acemel M., Palma A.J., Capitan-Vallvey L.F., Lopez-Villanueva J.A. (2014). Cantilever fabrication by a printing and bonding process. J. Microelectromech. Syst..

[111] Rivadeneyra A., Fernández-Salmerón J., Agudo-Acemel M., López-Villanueva J.A., Capitan-Vallvey L.F., Palma A.J. (2015). Improved manufacturing process for printed cantilevers by using water removable sacrificial substrate. Sens. Actuators A Phys..

[112] Cholleti E., Stringer J., Assadian M., Battmann V., Bowen C., Aw K. (2019). Highly stretchable capacitive sensor with printed carbon black electrodes on barium titanate elastomer composite. Sensors.

[113] Yao S., Zhu Y. (2014). Wearable multifunctional sensors using printed stretchable conductors made of silver nanowires. Nanoscale.

[114] Parrilla M., Cánovas R., Jeerapan I., Andrade F.J., Wang J. (2016). A textile-based stretchable multi-ion potentiometric sensor. Adv. Healthc. Mater..

[115] Parrilla M., Ferré J., Guinovart T., Andrade F.J. (2016). Wearable potentiometric sensors based on commercial carbon fibres for monitoring sodium in sweat. Electroanalysis.

[116] Gao W., Emaminejad S., Nyein H.Y.Y., Challa S., Chen K., Peck A., Fahad H.M., Ota H., Shiraki H., Kiriya D. (2016). Fully integrated wearable sensor arrays for multiplexed in situ perspiration analysis. Nature.

[117] Lee H., Choi T.K., Lee Y.B., Cho H.R., Ghaffari R., Wang L., Choi H.J., Chung T.D., Lu N., Hyeon T. (2016). A graphene-based electrochemical device with thermoresponsive microneedles for diabetes monitoring and therapy. Nat. Nanotechnol..

[118] Kim J., de Araujo W.R., Samek I.A., Bandodkar A.J., Jia W., Brunetti B., Paixão T.R., Wang J. (2015). Wearable temporary tattoo sensor for real-time trace metal monitoring in human sweat. Electrochem. Commun..

[119] Lisak G., Arnebrant T., Ruzgas T., Bobacka J. (2015). Textile-based sampling for potentiometric determination of ions. Anal. Chim. Acta.

[120] Seesaard T., Lorwongtragool P., Kerdcharoen T. (2015). Development of fabric-based chemical gas sensors for use as wearable electronic noses. Sensors.

[121] Kim Y.H., Kim S.J., Kim Y.-J., Shim Y.-S., Kim S.Y., Hong B.H., Jang H.W. (2015). Self-activated transparent all-graphene gas sensor with endurance to humidity and mechanical bending. ACS Nano.

[122] Gaspar C., Olkkonen J., Passoja S., Smolander M. (2017). Paper as active layer in inkjet-printed capacitive humidity sensors. Sensors.

[123] Sapsanis C., Buttner U., Omran H., Belmabkhout Y., Shekhah O., Eddaoudi M., Salama K.N. A nafion coated capacitive humidity sensor on a flexible pet substrate. Proceedings of the 2016 IEEE 59th International Midwest Symposium on Circuits and Systems (MWSCAS).

[124] Ali S., Hassan A., Hassan G., Bae J., Lee C.H. (2016). All-printed humidity sensor based on graphene/methyl-red composite with high sensitivity. Carbon.

[125] Romero F.J., Rivadeneyra A., Salinas-Castillo A., Ohata A., Morales D.P., Becherer M., Rodriguez N. (2019). Design, fabrication and characterization of capacitive humidity sensors based on emerging flexible technologies. Sens. Actuators B Chem..

[126] Rivadeneyra A., Salmerón J.F., Agudo-Acemel M., Capitan-Vallvey L.F., López-Villanueva J.A., Palma A.J. (2018). Asymmetric enhanced surface interdigitated electrode capacitor with two out-of-plane electrodes. Sens. Actuators B Chem..

[127] Bahoumina P., Hallil H., Lachaud J.-L., Abdelghani A., Frigui K., Bila S., Baillargeat D., Zhang Q., Coquet P., Paragua C. (2017). Chemical gas sensor based on a flexible capacitive microwave transducer associated with a sensitive carbon composite polymer film. Proceedings.

[128] Bahoumina P., Hallil H., Pieper K., Lachaud J., Rebière D., Dejous C., Abdelghani A., Frigui K., Bila S., Baillargeat D. Capacitive microwave sensor for toxic vapor detection in polluted environments. Proceedings of the 2017 IEEE Sensor.

[129] Kukkola J., Jansson E., Popov A., Lappalainen J., Mäklin J., Halonen N., Tóth G., Shchukarev A., Mikkola J.-P., Jantunen H. (2011). Novel printed nanostructured gas sensors. Proced. Eng..

